# Effects of microplastics and surfactants on surface roughness of water waves

**DOI:** 10.1038/s41598-023-29088-9

**Published:** 2023-02-03

**Authors:** Yukun Sun, Thomas Bakker, Christopher Ruf, Yulin Pan

**Affiliations:** 1 Department of Naval Architecture and Marine Engineering, University of Michigan, Ann Arbor, USA; 2 Department of Climate and Space Sciences and Engineering, University of Michigan, Ann Arbor, USA

**Keywords:** Physical oceanography, Fluid dynamics, Techniques and instrumentation

## Abstract

We study the flow physics underlying the recently developed remote sensing capability of detecting oceanic microplastics, which is based on the measurable surface roughness reduction induced by the presence of microplastics on the ocean surface. In particular, we are interested in whether this roughness reduction is caused by the microplastics as floating particles, or by surfactants which follow similar transport paths as microplastics. For this purpose, we experimentally test the effects of floating particles and surfactants on surface roughness, quantified by the mean square slope (MSS), with waves generated by a mechanical wave maker or by wind. For microplastics, we find that their effect on MSS critically depends on the surface area fraction of coverage. The damping by particles is observed only for fractions above *O* (5–10%), much higher than the realistic ocean condition. For surfactants, their damping effects on both mechanically generated waves and wind waves are quantified, which are shown to be much more significant than that by microplastics. Several new mechanisms/relations for roughness damping by surfactants are also identified. The implications of these experimental results to remote sensing are discussed.

## Introduction

Ocean plastics pollution is an urgent and global problem. An estimated eight million tons of plastics trash enters the ocean each year, and most of it is battered by sun and waves into microplastics. Information about the distribution and volume of microplastics is vital to address the removal of plastic pollution from the ocean environment. Recently the development of global observational systems for microplastics has been under active discussions among interdisciplinary communities^1–3^. Along this direction, it has been suggested^4,5^ that remote sensing techniques may be applied to track the microplastics concentrations in the ocean, through the detectable surface roughness suppression with the presence of microplastics. This idea of remote sensing was first implemented for the global ocean in^6,7^ with positive results identified. The principle of this implementation is to infer the microplastics concentration through the ocean surface roughness anomaly (i.e., lower-than-expected roughness) induced by microplastics, which can be accounted for by the difference between the real-time measurement from a spaceborne radar and a standard model of surface roughness.

**Figure 1:**
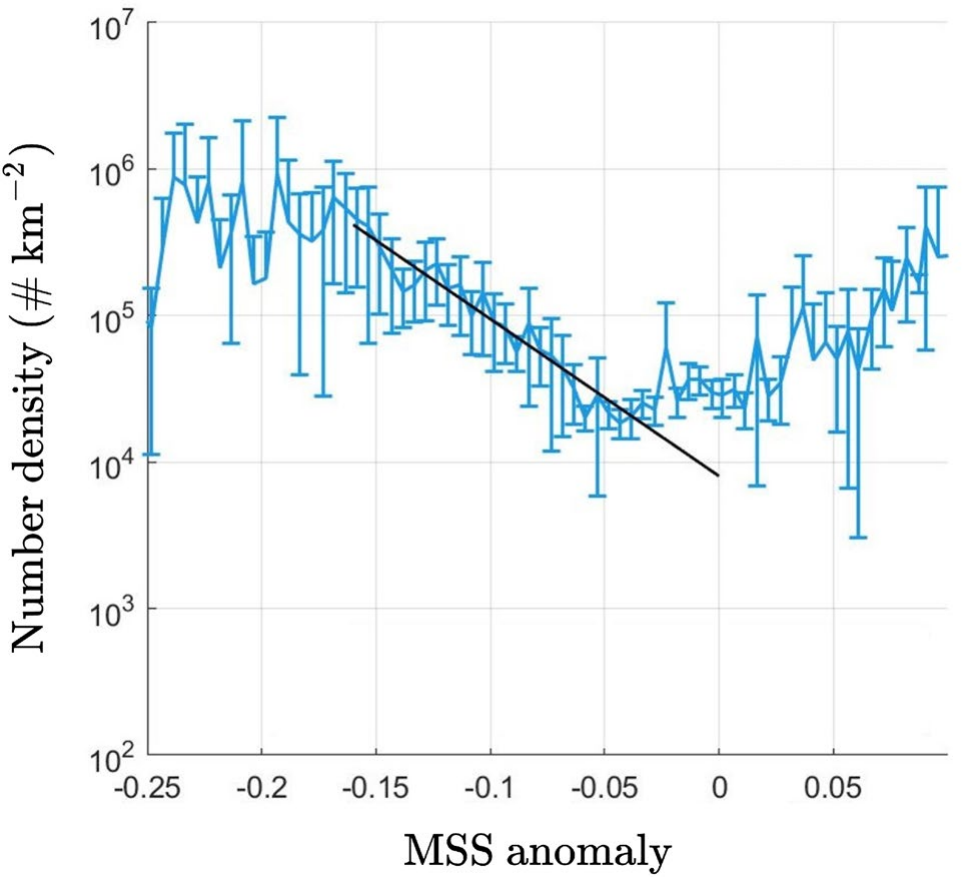
Relation between the MSS anomaly^7^ (computed from CYGNSS data and Katzberg model in the oceanic region within latitudes of $\pm 38^\circ$, and averaged from June 1, 2017 to May 31, 2018) and number density of microplasitics (computed by a global microplastic transport model^13^). The error bars represent $95\%$ confidence intervals with each one computed by data from the van Sebille model at locations with a given MSS anomaly. The range with a correlation between the MSS anomaly and number density is indicated ( ).

The new technique has been applied to data from the NASA CYGNSS (Cyclone Global Navigation Satellite System)^8,9^. Specifically, CYGNSS measures through the GPS L1 signal the normalized bi-static radar cross section (NBRCS), whose inverse provides the mean square slope (MSS) of the ocean surface, defined as

1 $$\begin{aligned} \text {MSS}=\int _0^{k_c} k^2 S(k) dk, \end{aligned}$$

where *k* is the wavenumber, *S*(*k*) is the omni-directional spectrum, and $k_c$ is the cut-off wavenumber depending on the incident angle and carrier wave frequency in remote sensing. For CYGNSS, $k_c$ takes an average value of 7.5 rad m$^{-1}$^10^. As defined by (1), $\text {MSS}\rightarrow \overline{\nabla \eta \cdot \nabla \eta }$ (variance of surface elevation gradient) for $k_c\rightarrow \infty$, and otherwise MSS quantifies the surface roughness up to a finite scale $k_c$. In addition to CYGNSS measurements, another source of MSS can be obtained by the standard Katzberg model^11^ which takes wind speeds from a NOAA reanalysis model^12^ as inputs. The MSS anomaly, defined as the relative difference between the CYGNSS measurements and Katzberg model results (normalized by the latter), is expected to account for the effect of microplastics on surface roughness, among other factors (such as error of the CYGNSS measurements and the influence of other physical processes). It is found^7^ that the MSS anomaly shows a favorable correlation with the concentration of oceanic microplastics computed from a global transport model, as shown in Fig. 1.

While this new technique shows promising applications, the underlying flow physics, in particular regarding the correlation between MSS anomaly and microplastics concentration, is not clear. It has been suggested^6^ that the correlation may be due to the enhanced damping of surface waves by microplastics as floating particles, referring to the earlier experimental results^14^. Indeed, in a sloshing wave tank, it is shown^14^ that the presence of layers of floating particles enhances the wave damping, with the enhancement increasing with the increasing number of particle layers. However, the experimental setup with the water surface fully covered by particles in a sloshing tank is not consistent with the situation of traveling ocean waves with microplastics only covering a small fraction of the surface. To represent the latter situation, experiments are needed to study the characteristics of surface waves in the presence of particles partially covering the free surface. Such experiments are currently not available.

Another hypothetical mechanism^7^ that can lead to the observed correlation is the wave damping effect by surfactants which share similar transport paths as microplastics^15^. Compared to a scarce number of studies on the effect of floating particles, there is a much larger body of literature on the effect of surfactants to surface waves. It has been observed in several field studies^16–22^ that the presence of surfactants on the ocean surface results in some damping effect of surface waves. Physically, the damping effect is believed to be caused by the Marangoni stresses (due to the inhomogeneous adsorption of surfactants at the interface) that can act in opposite directions of the wave motion^23^. In controlled experiments^24^ and numerical simulations^25,26^ where quantitative studies are possible, it is found that an optimal surfactant level associated with maximum damping exists for a given wave frequency. While these studies are conducted for different wave frequencies (in the range of 4 to 200 Hz) and identify different optimal surfactant levels, it is suggested^23^ that the optimal level may correspond to the situation of the surfactant-induced Marangoni wave having the same wavelength as the surface wave, leading to some resonance-like effect. For irregular waves as in the ocean, however, the effect of varying levels of surfactants is not sufficiently studied, and it is not clear whether an optimal level for wave damping exists for a wave spectrum. This question is also relevant to the correlation problem as such an optimal level implies a non-monotonic relation between concentration and MSS anomaly that somewhat contradicts the observed correlation.

For wind waves, experiments in wave tanks show that the presence of surfactants significantly suppresses the wave generation^27–31^. This phenomenon is usually explained through the reduction of wind shear stress by surfactants. With a surfactant level of $10^{-1}$ mol l$^{-1}$, it is experimentally shown^31^ that the shear stress can be reduced up to 30$\%$ compared to clean water for the same wind speed of 9.6 m s$^{-1}$. The minimum wind speed (and shear stress) to excite waves is also found to significantly increase with the presence of surfactants^28,30^. With surfactant level of $9.0\times 10^{-4}$ mol l$^{-1}$, the minimum wind speed leading to a wave growth is reported^27^ to be between 10 and 12.5 m s$^{-1}$. In spite of the scattered results, most studies focus on the wave characteristics at one (or only a few) surfactant levels. As a result, there are no systematic relations identified for the relevant parameters including wind speed, shear stress, wave growth rate, surfactant level and the associated surface tension. Uncovering these relations is not only helpful for interpretation/application of the aforementioned CYGNSS data, but also desirable in understanding wave generations on the ocean surface that is inevitably contaminated by surfactants.

In the present study, we aim to understand the mechanism of the MSS anomalies detected by CYGNSS as well as the general physics of wave damping by floating particles and surfactants. For these purposes, we conduct wave-tank experiments to test the effects of floating particles and surfactants to damp the waves generated by either a mechanical wave maker or wind. For floating particles, we use two sizes of particles with 0.5 cm and 0.8 cm (both in the range of oceanic microplastics) and consider their partial coverage of the free surface with varying area fraction. We find a critical dependence of the damping effect of particles on the area fraction, irrespective of the size of particles. The enhanced particle damping of MSS and energy of the surface waves is only observed for fractions above $O(5\sim 10\%)$. For low fraction of $O(0.1\%)$ that corresponds to real oceanic microplastics situations, the wave energy is not affected and the MSS is slightly increased (probably due to the diffraction by particles). For surfactants, results on mechanically generated waves show that the presence of surfactants damps the wave energy and MSS, but no optimal surfactant level (associated with maximum damping) can be identified as in the previous cases with monochromatic waves. The surfactants also suppress the wind-generated waves, leading to a higher critical wind speed to excite waves and a lower growth rate (for excited waves). Physically, we attribute this suppression to not only the previously argued effect of reduced wind shear stress, but also the wave damping by surfactants. Furthermore, at the same wind speed, we find that the wind shear stress depends exponentially on the surface tension, or depends on the concentration of surfactants in a power-law relation (with a negative exponent linearly correlated with the wind speed). In addition to the physical findings, the results confirm the presence of surfactant as the dominant contributor to the CYGNSS MSS anomaly, as suggested^4^ earlier. Some preliminary results of the paper are included in a report to the International Ocean Colour Coordinating Group (IOCCG)^32^

## Methods

### Facility and input parameters

**Figure 2:**
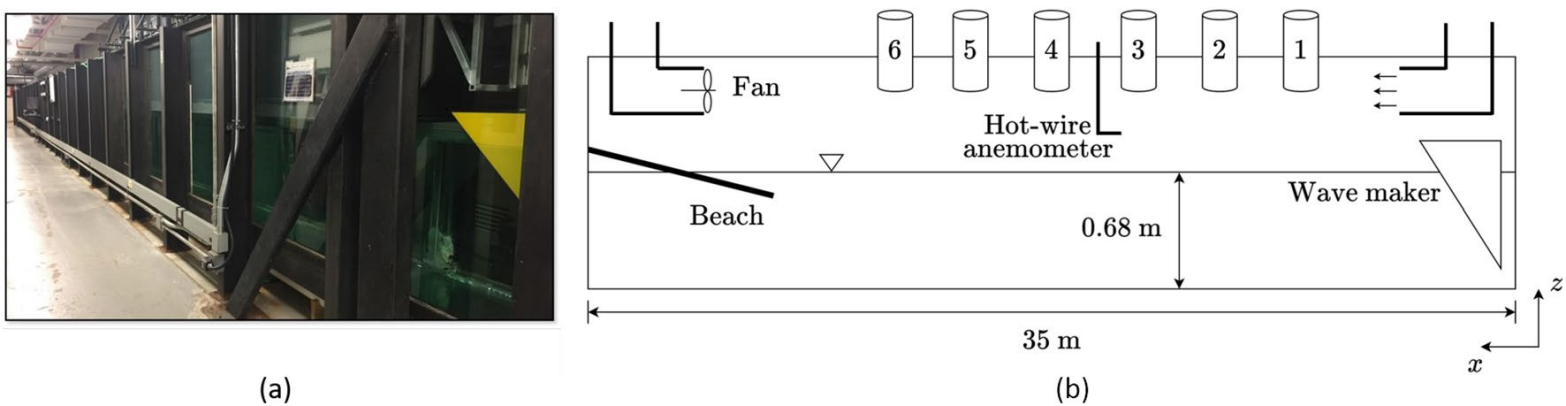
(**a**) A photograph of the wave tank; (**b**) a schematic sketch of the side view of the wave tank, including a wave maker, a wind tunnel, six ultrasonic sensors, a hot-wire anemometer, and a beach. The spacings between the sensors are not perfectly scaled in (**b**), with detailed information provided in Table 1.

The experiments are conducted in the wind-wave tank facility in the Marine Hydrodynamics Laboratory (MHL) at the University of Michigan, with a photograph and a schematic sketch of the tank shown in Fig. 2. The tank is 35 m long and 0.7 m wide with a water depth of 0.68 m. Waves can be generated by either a mechanical wave maker or wind through an open-loop tunnel, and are dissipated at one end of the tank by a beach with slope of $5^\circ$.

The wave maker is wedge-shaped with an angle of $30^\circ$ to the vertical direction, and spans the width of the wave tank. The motion of the wave maker is numerically actuated by a servo motor manufactured by Kollmorgen^®^ AKM2G. The servo motor is feedback controlled with a proportional-integral-derivative (PID) controller, according to an input frequency spectrum $S_\text {in}(f)$ that describes the power spectral density of the surface elevations $\eta (t)$. For an approximation of the real ocean scenario in this study, we use the Bretschneider spectrum, a two-parameter wind-wave spectrum empirically developed for fully-developed seas:

2 $$\begin{aligned} S_{\text {in}}(f) = \frac{1.25}{8\pi } \frac{f_p^4}{f^5} H_s^2 \textrm{e}^{-1.25\left( f_p/f\right) ^4}, \end{aligned}$$

where $f_p$ is the frequency of the peak mode and $H_s$ the significant wave height. In the current study, we use $f_p=1.25$ Hz, corresponding to $\lambda _p=1$ m as deep-water waves, and $H_s=3.9$ cm, corresponding to a moderate effective steepness $\epsilon =H_sk_p/2\approx 0.12$ with $k_p\equiv 2\pi /\lambda _p$ the peak wavenumber. In each experiment, the wave maker is actuated for 200 s, with acceleration and deceleration for 10 s at the start and the end of the actuation.

**Figure 3:**
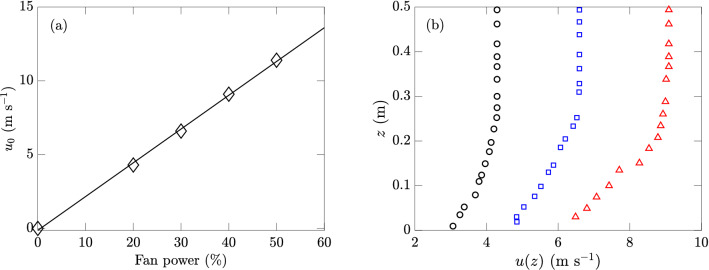
(**a**) Reference wind speeds $u_0$ for different fan powers ($\diamond$), with the linear fit (). (**b**) Wind profiles with fan powers of $20\%$ (), $30\%$ (), and $40\%$ (), correspondig to $u_0=4.29$, 6.59, and 9.09 m s$^{-1}$, respectively.

Winds of different speeds are generated in an open-loop wind tunnel, which is powered by a 40-hp fan and controlled by the percentage of its maximum output power. The bottom of the wind tunnel outlet is 10 cm above the water level. The wave tank is well sealed to prevent air leakage that may cause pressure fluctuations and therefore unsteady freestream wind speeds. A hot-wire anemometer manufactured by Extech^®^ is placed at 13 m downstream of the wind tunnel outlet to measure the wind speed. Operated at a sampling rate of 1 Hz, the anemometer is mounted on a vertical traverse that allows one-degree-of-freedom movements in the $z$-axis. Wind profiles are measured by adjusting the location of the traverse, with all measurements taken over the stationary state of the wind. It is found that the maximum wind speed along the vertical axis (hereafter the reference wind speed) satisfies a linear relation with the fan power, as shown in Fig. 3a. In the current study, we use three reference wind speeds of 4.29, 6.59, and 9.09 m s$^{-1}$ at fan powers of 20, 30, and $40\%$, with measured wind profiles plotted in Fig. 3b.

The data acquisition system consists of 6 Senix ToughSonic^®^ model-14 ultrasonic sensors mounted on the top of the wave tank. The intervals between the sensors are non-uniform, with distances from each sensor to the wave maker and the wind tunnel outlet listed in Table 1. The measurement of surface elevation $\eta (t)$ is converted from the voltage signals delivered by the sensors to a National Instruments^®^ data acquisition board. The sampling frequency of the sensors is fixed at 100 Hz (which is sufficient for the current study) although the maximum sampling frequency is up to 2000 Hz.

**Table 1:** Distances from the 6 wave sensors to the wave maker and to the wind outlet.

Sensor	1	2	3	4	5	6
Distance to wave maker (m)	9.35	11.38	14.23	18.66	22.76	28.45
Distance to wind outlet (m)	2.03	4.06	6.91	11.34	15.44	21.13

### Testing procedures

#### Experiments with microplastics

The major materials of floating plastic debris in real ocean are polyethylene and polypropylene^33^. In this study, we use two types of particles both made of polypropylene (density $\rho _p = 0.92$ kgm$^{-3}$): one as the PolyFil PolyPellets^®^ microbeads with irregular shape and average diameter $D_p\approx 0.5$ cm, and the other as McMaster Carr^®^ plastic balls with regular shape and diameter $D_p=0.8$ cm (both within the size range of the oceanic microplasitics^33^). To quantitatively test the wave damping by particles, we focus on mechanically generated waves, since the area fraction of particles (defined in (3)) is very difficult to control in wind waves (due to the drift by wind).

**Figure 4:**
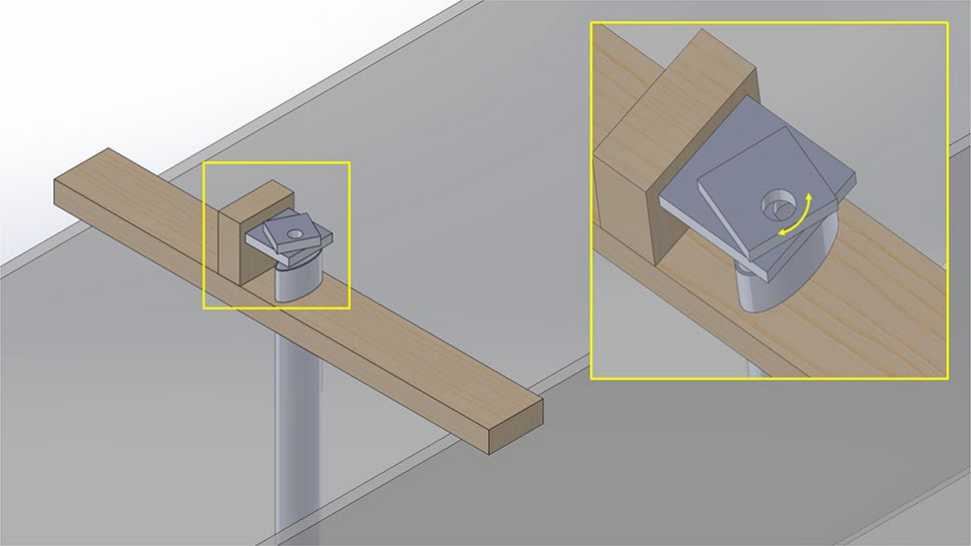
Device to drop the particles with an open-close mechanism operated at 1 Hz.

**Figure 5:**
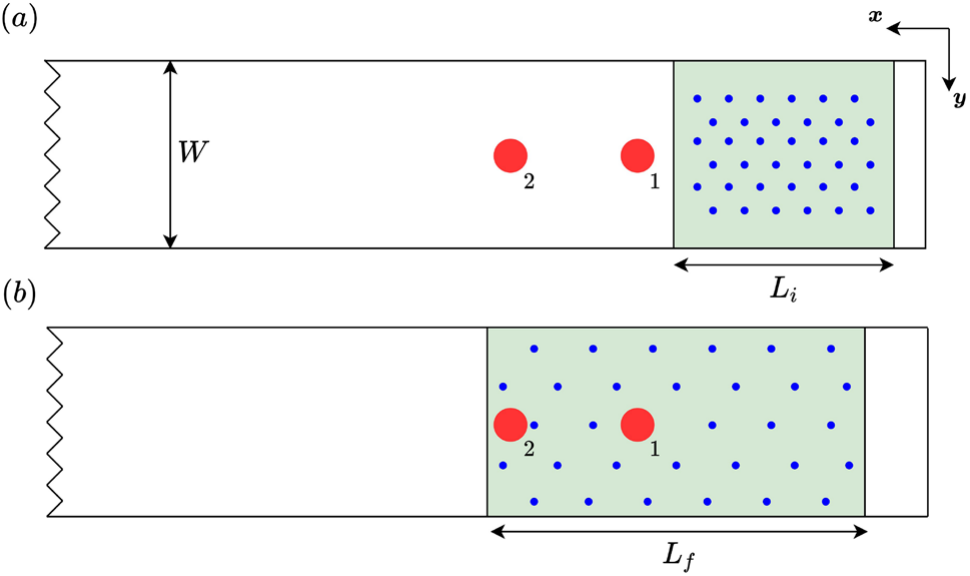
Schematic sketch of location and spread of particles at (**a**) the beginning and (**b**) the end of each experiment. For simplicity, only the locations of sensors 1 and 2 represented by ( ) are shown. The particle-covered regions are marked by green color with particles illustrated by ( ).

The experiments with particles in mechanically generated waves are conducted with the following procedures. We first place the particles in the wave tank at 8.53 m from the wave maker in calm water. Specifically, the particles are dropped into the wave tank through a circular pipe hanged vertically on the top of the tank (see Fig. 4). An open-close mechanism, actuated by a 20-kg digital servo motor operating at a frequency of 1 Hz and controlled by an Arduino Uno board, is used to maintain a constant number of particles dispatched. For particles of both sizes, the dispatch process is terminated until all particles are dropped into the wave tank. During the early stage of the dispatch process, the particles spread out radially at an almost constant speed. As more particles are dispatched, they fill the width of the tank and spread out both up- and downstream due to particle-particle and particle-surface interactions until they form a stationary, uniform, and single patch of particles with a recorded length $L_i$ (see Fig. 5a). As waves pass through the particles, they further spread out and drift downstream due to the wave effect (e.g., Stokes drift^15,34^), with the final spreading length (after waves pass through) recorded as $L_f$ (see Fig. 5b). To quantify the concentration of particles with varying spreading length, we define an (average) area fraction

3 $$\begin{aligned} C = \frac{N_p S_p}{W \overline{L}}, \end{aligned}$$

where $N_p$ is the number of particles, $S_p=\pi D_p^2/4$ is the planform area of one particle, *W* is the width of the tank and $\overline{L}=(L_i+L_f)/2$ is the average length of the spreading. In practice, we choose $N_p$ such that the value of *C* ranges from 0.1$\%$ to 20$\%$, with the lowest fraction close to the oceanic microplastics concentration. A total of 9 values of *C* in this range are tested (5 and 4 for smaller and larger particles), with each experiment repeated for 3 times to quantify the uncertainty level.

**Figure 6:**
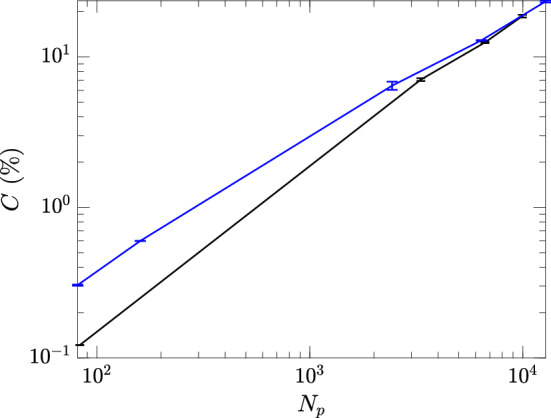
Values of *C* for particles with $D_p$=0.5 cm () and $D_p$=0.8 cm () at different $N_p$, with error bars representing one standard deviation on each side calculated from three repetitions.

To test the repeatability of using (3) to measure the concentration, we perform 3 repetitions for each value of $N_p$ for both types of particles, with the results shown in Fig. 6 including the error bars that represent one standard deviation on each side. Along with very small error bars (which illustrates sufficient repeatability), it appears that a power-law relation exists between *C* and $N_p$ for both types of particles and that the value of *C* is independent of the particle sizes for large $N_p$. While these observed behaviors need further confirmations, they are intriguing and may imply deeper physics. We leave the investigation of them to future work and focus on the effect of particles on surface waves in this study.

Finally, due to the drift of particles, the final length $L_f$ (for larger $N_p$) may cover the surface area beneath sensors 1 and 2. In order to robustly measure the effect of particles on waves (i.e., to avoid the acoustic reflection from particles and consistently consider waves after passing through all particles), we focus on sensor measurements downstream of the particle patch $L_f$. In addition, we exclude data from sensor 6 since it is relatively far away from the particles (so that the wave properties may be modified too much by nonlinear interactions instead of particle damping). Therefore, we focus on results based on sensors 3, 4 and 5 for floating particle experiments.

#### Experiments with surfactants

**Figure 7:**
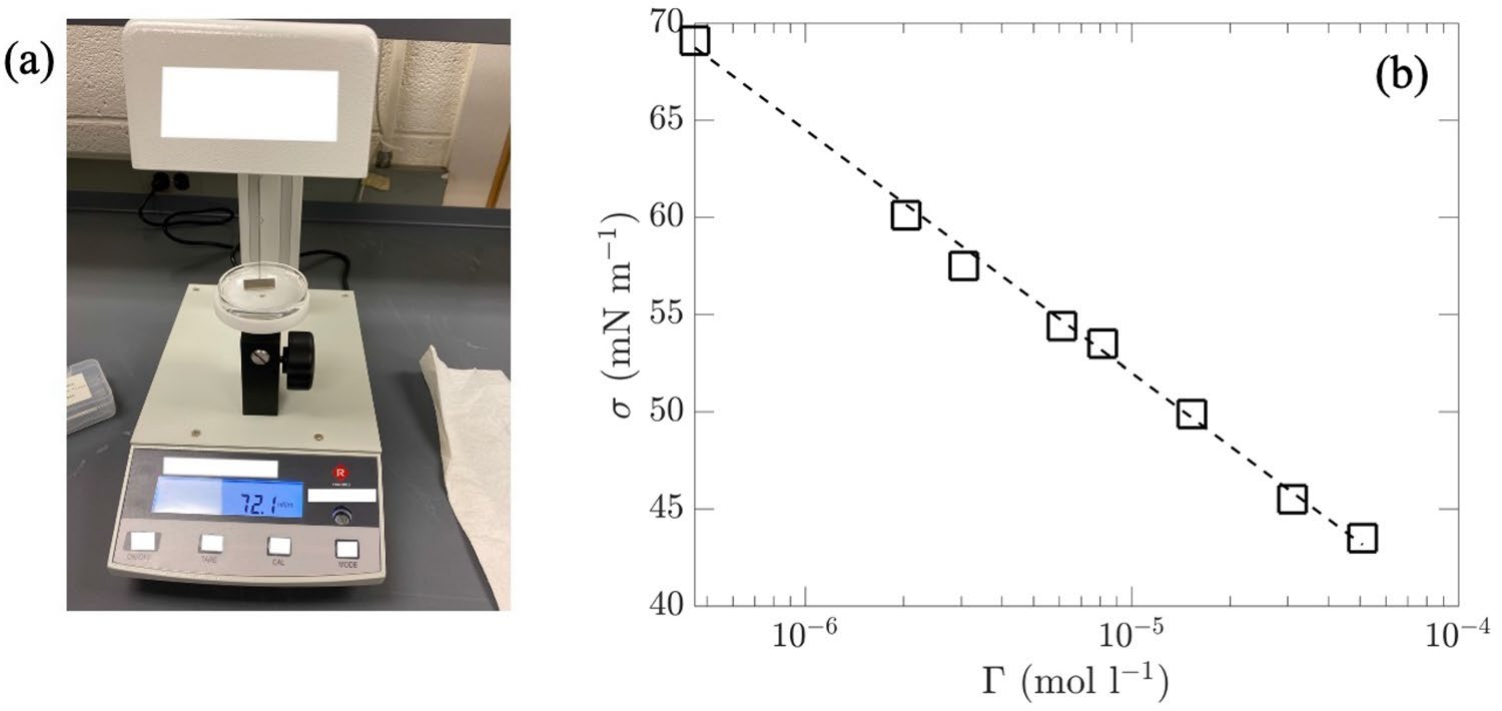
(**a**) The surface tensiometer BZY-101. (**b**) The measured surface tension $\sigma$ ($\square$) depending on the concentration $\Gamma$, with a logarithmic fit (${\varvec{\text{-- } \text{-- } \text{-- }}}$).

In the current study, we use a soluble surfactant, Triton X-100 (molecular weight 625 g mol$^{-1}$), that has been commonly used in many previous studies^24,31,35^. We consider nine concentrations $\Gamma = \Gamma _0 - \Gamma _8$ (in terms of mol l$^{-1}$) as listed in Table 2, with $\Gamma _0$, $\Gamma _1$, $\Gamma _2$, $\Gamma _6$, $\Gamma _7$, and $\Gamma _8$ tested for mechanically-generated waves, and $\Gamma _0\sim \Gamma _7$ tested for wind waves. The surface tension $\sigma$ for each concentration is measured from water samples by an Mxbaoheng^®^ BZY-101 surface tensiometer (Fig. 7a), which uses the Wilhelmy plate method based on the balancing of surface tension, gravitational, and buoyant forces on a platinum plate^36^. The measured surface tensions (with an error within $\pm 0.3$ mN/m for this device) are listed in Table 2 and plotted in Fig. 7b, showing a logarithmic relation with the value of $\Gamma$. All tested values of $\Gamma$ are below the critical micelle concentration (CMC) limit for Triton X-100, which is $\Gamma =23\times 10^{-5}$ mol l$^{-1}$ corresponding to a saturated surface tension $\sigma =30.6$ mN m$^{-1}$.

In each day, we perform a total of (maximum) 12 experiments related to one concentration $\Gamma$, that include three cases with different wind speeds and one case with the wave maker, with each repeated for three times. Between each two experiments we wait for 30 minutes, which allow the surfactants to reach a uniform distribution over the tank. At the end of each day more surfactants are added to the tank until the next level of desired concentration $\Gamma$ is reached. A circulation pump is turned on overnight to well mix the surfactants and water before the starting of experiments on the next day.

**Table 2:** Surfactant concentrations $\Gamma _0-\Gamma _8$ used in the experiments, with the corresponding values of surface tension $\sigma$.

Symbol	$\Gamma _0$	$\Gamma _1$	$\Gamma _2$	$\Gamma _3$	$\Gamma _4$	$\Gamma _5$	$\Gamma _6$	$\Gamma _7$	$\Gamma _8$
$\Gamma \times 10^{5}$ (mol l$^{-1}$)	0	0.05	0.20	0.31	0.61	0.81	1.53	3.11	5.09
$\sigma$ (mN m$^{-1}$)	72.0	69.1	60.1	57.5	54.4	53.5	49.9	45.5	43.5

Finally, we summarize all experiments conducted for microplastics and surfactants in Table 3.

**Table 3:** Summary of setups for experiments with microplastics and surfactants.

Particles/surfactants	Concentration	Wave generation	Repetitions
$D_p\approx 0.5$ cm	$C=0.12$, 7.07, 12.48, and $18.69\%$	Mechanical	3
$D_p=0.8$ cm	$C=0.30$, 0.60, 6.44, 12.88, and $23.55\%$	Mechanical	3
TX-100	$\Gamma _0$ to $\Gamma _2$ and $\Gamma _6$ to $\Gamma _8$	Mechanical	3
TX-100	$\Gamma _0$ to $\Gamma _7$	Wind	3

### Quantities of interest

**Figure 8:**
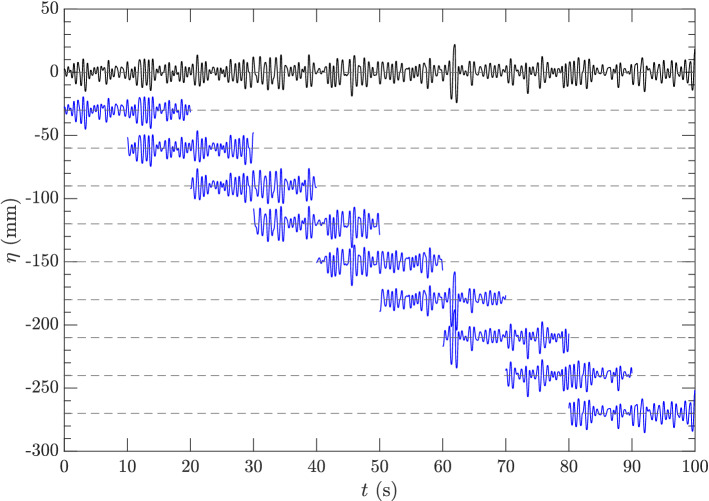
A typical time series $\eta (t)$ () and 9 segments (), with data taken from sensor 1 in clean water test with mechanically generated waves. Each segment is vertically shifted for better visualization with the mean water level indicated by (${\varvec{\text{-- } \text{-- } \text{-- }}}$).

Frequency spectra of surface elevations are calculated from the time series measured by the sensors in a time interval of 100 s of stationary state (in time). We use a standard way^37^ to compute the spectra as an ensemble average over spectra of 9 segments of the time series with $50\%$ overlapping of each two segments (see Fig. 8). For each segment ($\Delta t=20$ s with 2000 data points), we use a Tukey window function to taper the tails of the segment, and evaluate the spectrum as

4 $$\begin{aligned} S_f(f) = (\Delta t/2) \left| \hat{\eta }(f)\right| ^2, \end{aligned}$$

with $\hat{\eta }(f)$ the coefficient of cosine Fourier series of the tapered segment.

Given $S_f(f)$, we are interested in two quantities of the mean square slope (MSS) and the wave energy (*E*), defined respectively by (1) and

5 $$\begin{aligned} E = \int _0^{k_c} S(k) \,dk, \end{aligned}$$

with $S(k)=gS_f(f)/(8\pi ^2f)$. While the evaluation by (5) is not sensitive to the cutoff wavenumber $k_c$ for $k_c$ above 7.5 rad m$^{-1}$ (correponding to CYGNSS applications), the value of MSS evaluated by (1) depends more significantly on $k_c$ up to *O*(1000) rad m$^{-1}$. This dependence will also be discussed later in the context of the implications of the experimental results to CYGNSS applications.

## Reference results in clean water

In this section, we present the reference results in clean water, with the purpose of characterizing the properties, especially quantifying the uncertainty levels, of the results.

### Wave maker experiments

**Figure 9:**
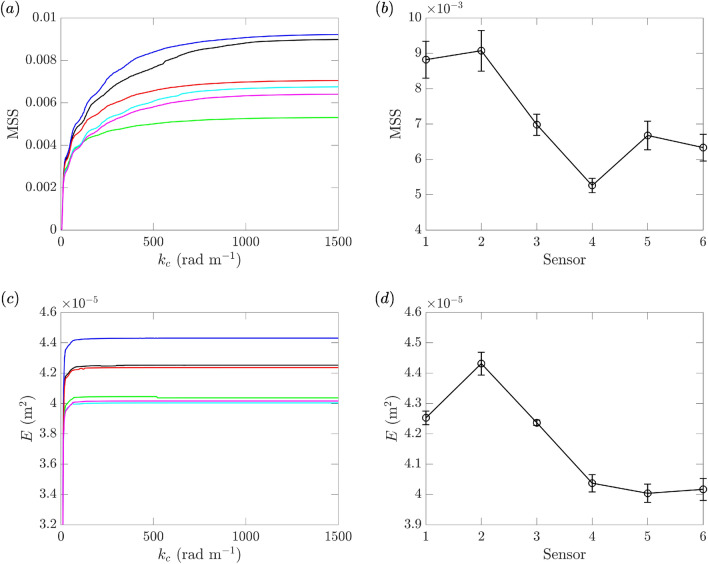
(**a**) MSS and (**c**) *E* for varying $k_c$ at sensor 1 (), 2 (), 3 (), 4 (), 5 (), and 6 () in a typical clean-water experiment. (**b**) MSS and (d) *E* with $k_c=1000$ rad m$^{-1}$ at 6 sensors, including the mean values () and error bars as one standard deviation on both sides, calculated over 10 repetitions in one day.

We first investigate the results for mechanically generated waves, with Fig. 9a and c showing typical results of MSS and *E* as functions of $k_c$ measured by the six sensors in one of the runs. It can be seen that the values of MSS and *E* converge for $k_c$ above $\mathcal {O}(10^3)$ rad m$^{-1}$ and $\mathcal {O}(10)$ rad m$^{-1}$, respectively. The main results of the paper will be presented with $k_c$=1000 rad m$^{-1}$ for both quantities (a lower value of $k_c$ results in inaccurate calculation in terms of (1) while a higher value results in higher uncertainty among repetitions of the experiments). Figure 9b and d show the mean values and error bars (as one standard deviation on both sides) of MSS and *E* with $k_c$=1000 rad m$^{-1}$, computed from 10 repetitions of each experiment within one day. For this value of $k_c$, the errors of MSS and *E* are both acceptable (with this $k_c$ for MSS reflecting a balance between the accuracy of (1) and uncertainties in repetitions), although the uncertainty levels of MSS are generally larger especially for sensor 2 in this case. This larger uncertainty of MSS is mainly caused by the noise in the high-frequency motion of the wave maker (that is not precisely controlled due to the relatively low-frequency implementation of the feedback control), which is amplified due to the $k^2$ dependence in the MSS calculation (1). We note that both MSS and *E* can increase toward downstream over two adjacent sensors (say MSS for sensors 4 and 5, *E* for sensors 1 and 2). These are due to the nonlinear wave effect which may transfer energy to high wavenumbers (leading to an increased MSS) or exchange energy among linear and nonlinear parts (leading to an increased *E* as the linear part of the energy).

We also remark that the MSS measured over different days has a higher uncertainty level compared to that shown in Fig. 9b (which is quantified within one day). This is probably due to the different levels of impurities (e.g., organic materials) that are flushed into the tank during the cleaning procedure at the start of each day (which is difficult to avoid for tank of this size), as well as the background noise (e.g., the wind-wave tank is located nearby a 109.7 m long towing tank which is under different operations each day). These factors may have larger influence to the higher wavenumber component of the spectrum resulting in higher uncertainty in MSS. In order to avoid such uncertainty introduced by cross-day experiments, we compare our results with and without microplastics (with multiple repetitions for each) within the same day to quantify the effect of microplastics.

### Wind wave experiments

**Figure 10:**
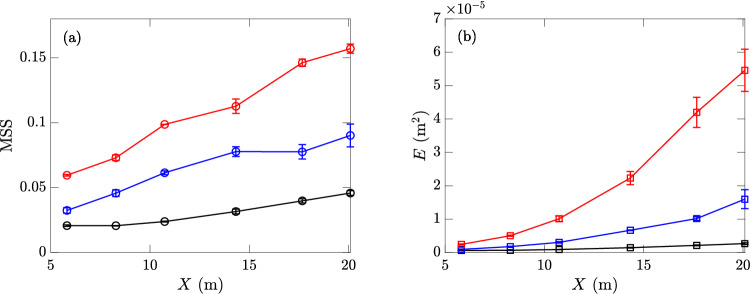
(**a**) MSS and (**b**) *E* with $k_c=1000$ rad m$^{-1}$ at 6 sensors, generated by reference wind speeds of 4.29 (), 6.59 (), and 9.09 () m s$^{-1}$, including the mean values and error bars as one standard deviation on both sides, calculated over 10 repetitions in one day.

The MSS and energy *E* measured in wind wave experiments at three different reference wind speeds are shown in Fig. 10a and b, as functions of the fetch (measured by the locations of sensors 1–6). The error bars are reasonably small for both quantities, indicating the robustness in the systems of wind-wave generation and measurement. For energy *E*, the uncertainty is higher than the cases with mechanically generated waves mainly because of the variation close to the wind-generated peak mode located at the relatively low frequency range of the spectrum (so it mainly adds uncertainty to *E* but not MSS). It is also clear that both MSS and *E* increase with the fetch and wind speeds, consistent with previous results^16,38–41^. The condition of fully-developed sea cannot be achieved in the current 35-m tank for the three operational wind speeds.

## Results of experiments with floating particles

**Figure 11:**
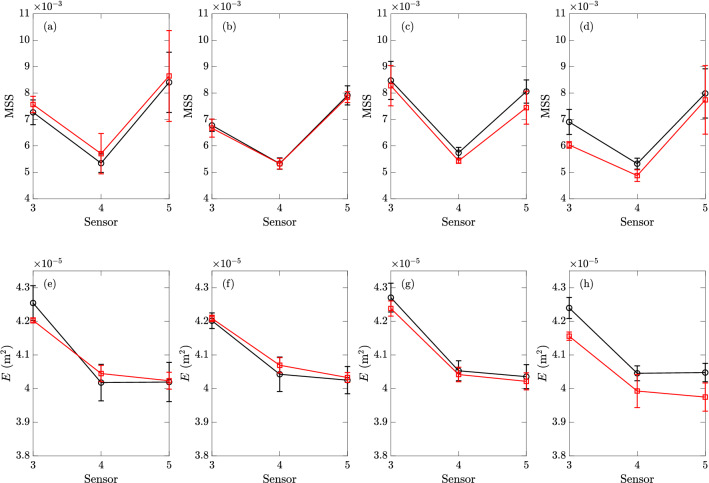
The MSS (, top row) and energy *E* (, bottom row) measured in experiments with particles of size $D_p\approx 0.5$ cm by sensors 3 to 5, with area fractions of (**a**)(**e**) $0.12\%$, (**b**)(**f**) $7.07\%$, (**c**)(**g**) $12.48\%$, and (**d**)(**h**) $18.69\%$, together with the clean-water results () (obtained in the same day as particle experiments) for reference. The error bars of all results correspond to one standard deviations on both sides of the mean values.

**Figure 12:**
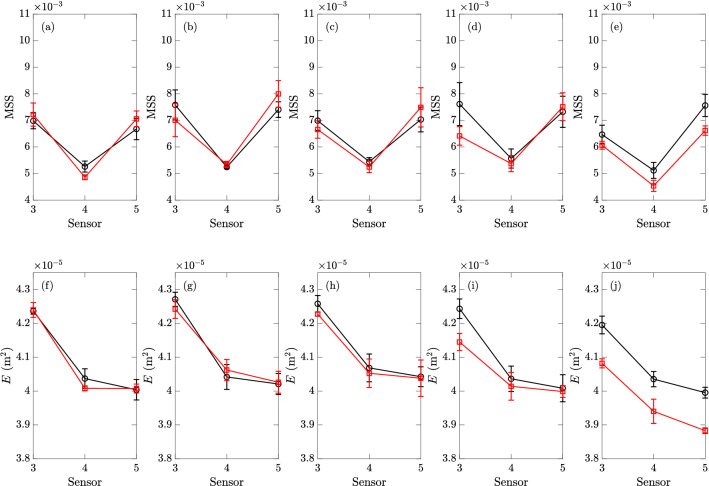
The MSS (, top row) and energy *E* (, bottom row) measured in experiments with particles of size $D_p=0.8$ cm by sensors 3 to 5, with area fractions of (**a**)(**f**) $0.30\%$, (**b**)(**g**) $0.60\%$, (**c**)(**h**) $6.44\%$, and (**d**)(**i**) $12.88\%$, and (**e**)(**j**) $23.35\%$, together with the clean-water results () (obtained in the same day as particle experiments) for reference. The error bars of all results correspond to one standard deviations on both sides of the mean values.

The values of MSS and *E* at sensors 3 to 5 with small ($D_p\approx 0.5$ cm) and large ($D_p=0.8$ cm) particles are presented respectively in Figs. 11 and 12, along with the clean-water results. The mean values and error bars in each sub-figure are computed from 3 repetitions of experiments with particles (with corresponding size and area fraction *C*), as well as 10 repetitions of clean-water experiments within the same day for reference (or comparison). In general, we find that the damping effects for MSS and *E* only become obvious for sufficiently large value of *C* for both particle sizes, e.g., in Fig. 11d,h,e,j, where we see smaller values of the two quantities in experiments with particles relative to the clean-water experiments. For small values of *C*, the MSS and energy *E* are barely affected for either particle size, in terms of the average values over the three sensors compared to the clean-water results (cf. Fig. 11b,f,b,g). The only exception to this general behavior is the MSS at small area fraction *C* especially for the small particle (Fig. 11a), which shows higher values at all sensors compared to the clean-water results. This is probably caused by the diffraction of short waves by the particles (or a patch of particles) that tends to increase the MSS especially when the particles are smaller. On the other hand, the MSS at small area fraction *C* for larger particles shows non-uniform variations among the three sensors, likely due to the particle-disturbed (but not damped) wave spectrum which evolves as the waves travel downstream.

**Figure 13:**
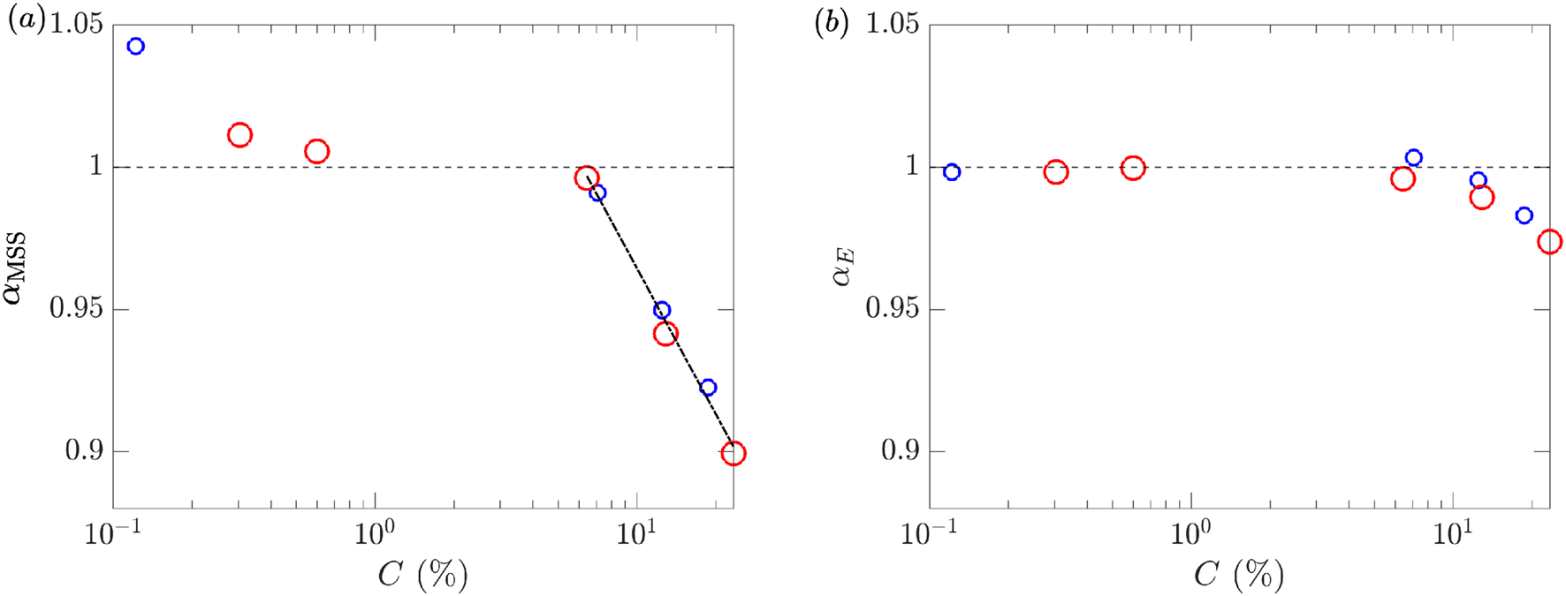
(**a**) $\alpha _{{\rm MSS}}$ and (**b**) $\alpha _E$ as functions of area fraction *C* for particle sizes $D_p\approx 0.5$ cm () and $D_p=0.8$ cm (). The reference value of $\alpha _{ {\rm MSS}}=1$ and $\alpha _E=1$ (i.e., no effect from particles) are indicated (${\varvec{\text{-- } \text{-- } \text{-- }}}$). A logarithmic fit to $\alpha _{ {\rm MSS}}$ at large values of *C* is shown (- $\cdot$ -).

Since the results measured by a single sensor may be subject to higher uncertainty and influenced by other physical processes (e.g., different nonlinear effects introduced after the modification of spectra by the presence of particles) in addition to the particle damping, we further quantify the effects of particles by examining the average quantities:

6 $$\begin{aligned} \overline{ {\rm MSS}}_{ij} = \frac{1}{j-i+1} \sum _{n=i}^j {\rm MSS}_n, \quad \overline{E}_{ij} = \frac{1}{j-i+1} \sum _{n=i}^j E_n, \quad \text {for } j\ge i, \end{aligned}$$

where $\text {MSS}_n$ and $E_n$ are MSS and *E* measured at sensor *n*. For results in this section, we use $i=3$ and $j=5$ to obtain average values of $\overline{\text {MSS}}_{35}$ and $\overline{E}_{35}$. We further define the ratio $\alpha$ to quantify the effect of particles relative to the clean-water results, as

7 $$\begin{aligned} \alpha _\phi = \overline{\phi }_{35}^p/\overline{\phi }_{35}^{c}, \end{aligned}$$

where $\phi$ represents MSS or *E*, and the superscripts *p* and *c* denote results with the presence of particles and in clean water respectively. The quantities $\alpha _{ {\rm MSS}}$ and $\alpha _E$ are presented in Fig. 13 as functions of the area fraction *C* for both particle sizes. We observe that, when measured by the area fraction, the results obtained with the two particle sizes almost collapse to a single curve, indicating that the effects of particles on surface waves are irrespective of the particle size in the test range. The damping effects for both quantities become evident for values of *C* above $C^*\sim O(5-10\%)$. The effect of particles on MSS is much larger than their effect on *E* (e.g., at the highest value of *C*, $\alpha _{ {\rm MSS}}=0.90$ but $\alpha _E=0.97$), suggesting that the damping effect of the tested particles focuses more on the short waves. Finally, for higher values of *C*, the damping effect for MSS seems to exhibit a logarithmic relation with $\alpha _{ {\rm MSS}}-1 \sim -\log (C/C^*)$ over half a decade indicated by the data in Fig. 13a.

**Figure 14:**
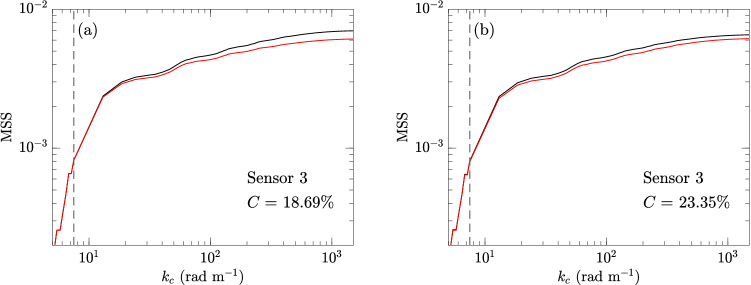
MSS computed with different $k_c$ with (**a**) particles of $D_p\approx 0.5$ cm, $C=18.69\%$ () and (**b**) particles of $D_p=0.8$ cm, $C=23.35\%$ (). The reference clean-water results (), as well as the indication of $k_c=7.5$ rad m$^{-1}$ (${\varvec{\text{-- } \text{-- } \text{-- }}}$), are shown in both (**a**) and (**b**).

Before concluding the section on floating particles, we briefly discuss the implication of the results to the remote sensing (e.g., CYGNSS) applications. Figure 14 shows a typical result of MSS as a function of $k_c$ measured by sensor 3 for both sizes of particles at their highest area fraction *C*, along with the reference results in clean water. It is clear from the plots that for $k_c=7.5$ rad m$^{-1}$ (corresponding to CYGNSS application), the effect of particles on MSS is negligible even for the highest concentration (that is much higher than the oceanic concentration). At realistic oceanic concentrations, the MSS is not meaningfully damped up to $k_c=1000$ rad m$^{-1}$, as evidenced from Fig. 13. Therefore, the results are sufficient for us to conclude that the MSS anomalies observed by CYGNSS are not caused by the effects of microplastics as floating particles.

## Results of experiments with surfactants

### Results for mechanically generated waves

**Figure 15:**
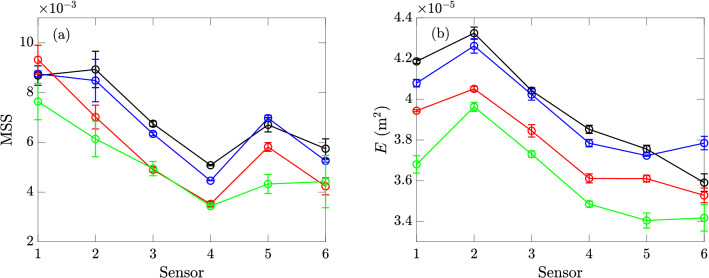
(**a**) MSS and (**b**) *E* measured by different sensors in experiments of mechanically generated waves with $\Gamma =\Gamma _0$ and $\sigma =72.0$ mN m$^{-1}$ (), $\Gamma =\Gamma _1$ and $\sigma =69.1$ mN m$^{-1}$ (), $\Gamma =\Gamma _7$ and $\sigma =45.5$ mN m$^{-1}$ (), and $\Gamma =\Gamma _8$ and $\sigma =43.5$ mN m$^{-1}$ (). The error bars represent one standard deviation on each side of the mean value computed from 3 repetitions.

**Figure 16:**
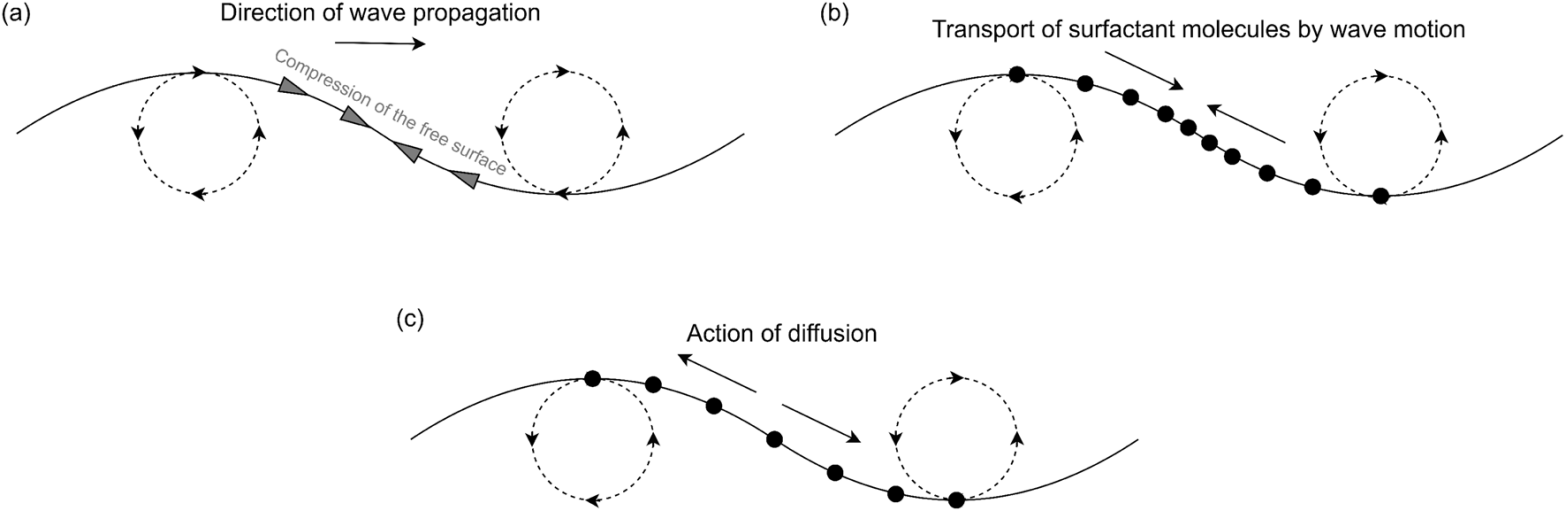
(**a**) Compression of the free surface by the wave motion; (**b**) transport of surfactant molecules to create the density gradient; (**c**) surface diffusion process to remove the density gradient.

Figure 15 shows values of MSS and *E* measured by all six sensors at different surfactant concentrations $\Gamma$ (leading to different surface tensions $\sigma$). We observe significant damping of both MSS and *E* with the presence of surfactants, compared to the results in clean water (i.e., $\Gamma =\Gamma _0$ and $\sigma =72.0$ mN m$^{-1}$). Another interesting phenomenon seen in Fig. 15 is that the major surfactant-induced damping effect of MSS and energy *E* occur in a short distance from the wave maker, namely before sensor 2 (15.47 m) for MSS and before sensor 1 (13.00 m) for *E*. After this short distance, the surfactant-induced damping effect becomes insignificant, i.e., the values of MSS and *E* do not further deviate from the results in clean water. This phenomenon suggests a hypothetical mechanism that for each surfactant concentration, there might exist a critical level of wave amplitude below which there is no enhanced damping by surfactants. This hypothetical mechanism, although yet to be verified, can be further elaborated as follows: The compression/expansion of the free surface by the wave motion (Fig. 16a) induces surface transport of the surfactant molecules to create gradients of their concentration (Fig. 16b). The transport happens together with a surface diffusion process (Fig. 16c) and an adsorption/desorption process^23^ with both removing the surfactant density gradient. The Marangoni damping (resulted from the reverse Marangoni convection due to the gradient of surface tension) can be suppressed if the wave steepness is sufficiently small so that the compression/expansion-induced reverse Marangoni convection occurs slower than the diffusion or the adsorption/desorption process.

**Figure 17:**
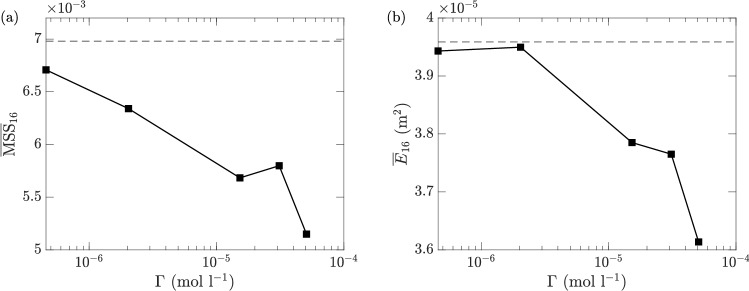
(**a**) $\overline{\text {MSS}}_{16}$ and (**b**) $\overline{E}_{16}$ at different concentration levels of the surfactants. The results for $\Gamma =\Gamma _0$ (i.e., clean water) are indicated by (${\varvec{\text{-- } \text{-- } \text{-- }}}$) in both sub-figures.

We are also interested in the dependence of surfactant-induced damping on the concentration levels. For this purpose, we plot $\overline{\text {MSS}}_{16}$ and $\overline{E}_{16}$ as functions of the concentration $\Gamma$ in Fig. 17. These results are remarkably different from the previous experiments/simulations based on a monochromatic wave (with frequencies both larger and smaller than the peak frequency in (2))^24–26^, where an optimal concentration level exists corresponding to the maximum reduction rate for each wave frequency. Nevertheless, for irregular waves as in our study, the relation between the damping effect and the concentration level is not monotonic, with $\overline{\text {MSS}}_{16}$ showing a local maximum at $\Gamma =\Gamma _7$ and $\overline{E}_{16}$ at $\Gamma =\Gamma _2$ (we remark that these local maxima may change for different input wave spectra). These complicated behaviors indicate that to further quantitatively understand the enhanced damping effect of surfactant to irregular ocean waves, one needs to consider the Marangoni damping in the presence of nonlinear wave interactions among a range of wave frequencies.

**Figure 18:**
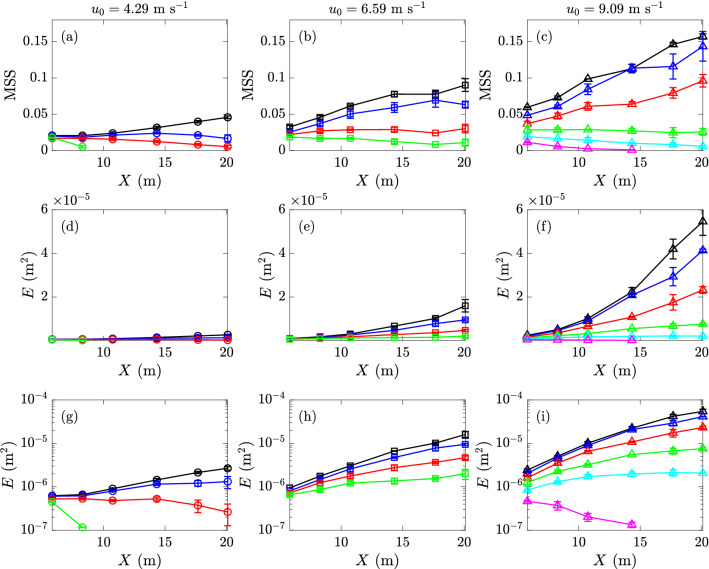
MSS and *E* with $\Gamma =\Gamma _0$ and $\sigma =72.0$ mN m$^{-1}$ (), $\Gamma =\Gamma _1$ and $\sigma =69.1$ mN m$^{-1}$ (), $\Gamma =\Gamma _2$ and $\sigma =60.1$ mN m$^{-1}$ (), $\Gamma =\Gamma _3$ and $\sigma =57.5$ mN m$^{-1}$ (), $\Gamma =\Gamma _4$ and $\sigma =54.4$ mN m$^{-1}$ (), and $\Gamma =\Gamma _5$ and $\sigma =53.5$ mN m$^{-1}$ () at fetches *X* corresponding to sensors 1 to 6. The top and middle rows plot the MSS and *E* respectively in a linear-linear scale, and the bottom row plots *E* in a linear-logarithmic scale. The columns from left to right are for reference wind speeds of 4.29, 6.59, and 9.09 m s$^{-1}$, respectively.

### Results for wind waves

Figure 18 plots the MSS and energy *E* as functions of the fetch *X* for different wind speeds and different concentration levels of surfactant. We first note that for cases with sufficiently low wind speed and/or high surfactant concentration, there exist sensor measurements at a similar signal level to the measurements in calm water. These data points (with a criterion of *E* less than 1.5 times that of noises measured in calm water) are physically considered as cases of no wave generation and are practically excluded in Fig.  18. It is also noted that in Fig. 18 there are some curves which decrease with the increase of *X* as well as “broken” lines (say in (a), (c) and (g)), indicating wave growth at shorter fetch but then decay at longer fetch. This phenomenon is due to the convection of surfactant downstream by wind, which results in a higher concentration downstream associated with stronger damping effect.

**Figure 19:**
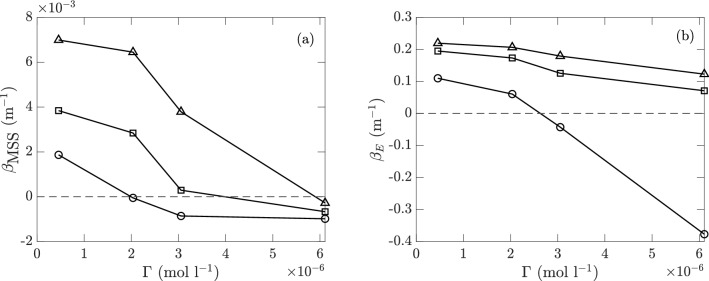
(**a**) Linear growth rate $\beta _{ {\rm MSS}}$ for MSS (with MSS $\sim \beta _{ {\rm MSS}}X$) and (**b**) exponential growth rate $\beta _E$ for *E* (with $E \sim \exp (\beta _E X)$) for varying surfactant concentrations $\Gamma$ at reference wind speeds $u_0=4.29$ (), 6.59 (), and 9.09 m s $^{-1}$ (). Only cases with wave excitation are included, and negative values indicate wave decay from sensor 1 to 6. The levels of $\beta _{ {\rm MSS}}=0$ and $\beta _E=0$ are marked (${\varvec{\text{-- } \text{-- } \text{-- }}}$).

From Fig. 18 we find that MSS and energy *E* show respectively a linear growth (cf. (a–c)) and an exponential growth (cf. (d–i)) with fetch *X*, especially for the initial stage (i.e., short fetch) far before the fully-developed condition (i.e., dissipation much weaker than growth). While the exponential growth of energy *E* is consistent with previous results^27,42,43^, we are not aware of any previous finding regarding the linear growth of MSS. These results indicate that in a wind sea, the peak mode grows much faster than the high-wavenumber portion of the spectrum in the initial stage of development. The linear growth rate $\beta _{ {\rm MSS}}$ for MSS (with MSS $\sim \beta _{ {\rm MSS}}X$) and exponential growth rate $\beta _E$ for *E* (with $E \sim \exp (\beta _E X)$) are fitted from data and summarized in Fig. 19, both as functions of $\Gamma$ for each reference wind speed (only cases with wave excitation are shown). We observe that higher surfactant concentrations consistently reduce the growth rates $\beta _{ {\rm MSS}}$ and $\beta _E$.

**Figure 20:**
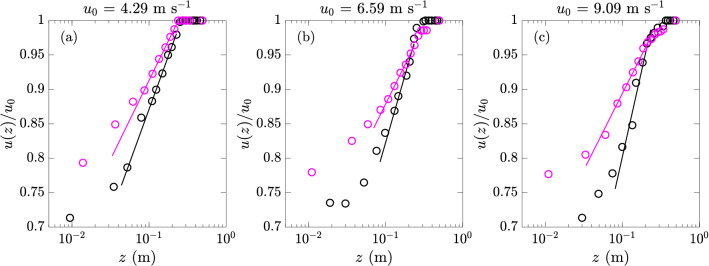
Wind profiles $u(z)/u_0$ with surfactant concentrations $\Gamma _0$ ($\circ$) and $\Gamma _4$ (), with linear fittings () and (), at reference wind speeds (**a**) $u_0=4.29$, (**b**) 6.59, and (**c**) 9.09 m s$^{-1}$.

To understand the mechanisms underlying the effect of surfactants on MSS and *E*, we first investigate the wind shear stress under different levels of concentration. For this purpose, we follow the procedure^44^ to extract the friction velocity $u_*$ (and thus shear stress $\tau =\rho u_*^2$) from the logarithmic layer of the wind profile, defined as

8 $$\begin{aligned} u(z) = \frac{u_*}{\kappa } \ln {\frac{z}{z_0}}, \end{aligned}$$

where $\kappa =0.41$ is the von Kármán constant and $z_0$ is a roughness parameter (characterizing the viscous length scale or thickness of viscous sub-layer). Figure 20 shows some typical wind profiles as well as the fitting using $u(z)=A\ln {z} + B$ from which the friction velocity $u_*=\kappa A$ and wind shear stress $\tau$ can be calculated.

**Figure 21:**
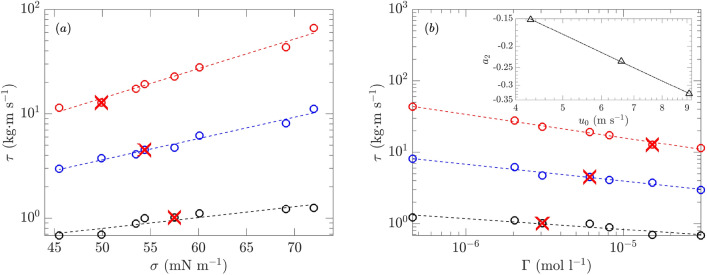
Wind shear stress $\tau$ as a function of (**a**) $\sigma$ and (**b**) $\Gamma$ at $u_0=4.29$ ($\circ$), 6.59 (), and 9.09 m s$^{-1}$ (), with exponential and power-law fits marked by (${\varvec{\text{-- } \text{-- } \text{-- }}}$) for each $u_0$. Critical points of $\sigma _c$, $\Gamma _c$, $\tau _c$ corresponding to threshold of wave excitation are marked by  for each $u_0$. Values of $a_2$ ($\triangle$) at different $u_0$ with linear fit (- $\cdot$ -) are shown in the inset of sub-figure (**b**).

The wind (shear) stress $\tau$ for varying surfactant concentrations (thus varying surface tensions) is plotted in Fig. 21 for all three reference wind speeds $u_0$. For each wind speed we mark the critical point ($\Gamma _c, \sigma _c, \tau _c$) corresponding to the boundary below which no waves are excited. It is clear that for cases both above and below the critical points, the wind stress shows an exponential dependence on surface tension ($\tau \sim \exp (a_1\sigma )$) and a power-law dependence (which can be expected from Fig. 7b) on the surfactant concentration level ($\tau \sim \Gamma ^{-a_2}$) at each same wind speed. The inset of Fig. 21b plots the values of $a_2$ for different reference wind speeds $u_0$, which suggests a linear relation of $a_2 \sim u_0$ implying also $a_1 \sim u_0$ (although more data at other wind speeds may be needed to further consolidate this relation).

In addition, the critical points ($\Gamma _c, \sigma _c, \tau _c$) in Fig. 21 reveal interesting physics on wind-wave generation. While higher wind stress implies stronger tendency for wave generation, it is possible, as suggested by Fig. 21, that waves are excited at lower $\tau$ (say $u_0=4.29$ m s$^{-1}$ and $\sigma$ around 70 mN m$^{-1}$) but suppressed at higher $\tau$ (say $u_0=6.59$ m s$^{-1}$ and $\sigma$ around 50 mN m$^{-1}$). This fact is only possible if the latter is subject to higher damping effect (which overcomes the higher wind stress) due to higher concentration of surfactants. Therefore, to understand wave generation in the presence of surfactants, it is important to consider the surfactant-induced damping as an additional physical factor. For cases in clean water with a monochromatic wave profile (for which damping can be clearly quantified), it has been demonstrated by numerical simulations^43^ that the wave growth rate uniquely depends on wind stress above some balanced value (which characterizes the critical state of growth from wind stress balancing the damping). The situation for our case, however, is significantly more complicated, as the damping effect depends on both the surfactant concentration level and wind speed (which affect the wavelength of the peak mode to be excited that in turn changes the damping by surfactants). These dependence relations need to be further investigated in order to fully understand the wind-wave generation in the presence of surfactants.

**Figure 22:**
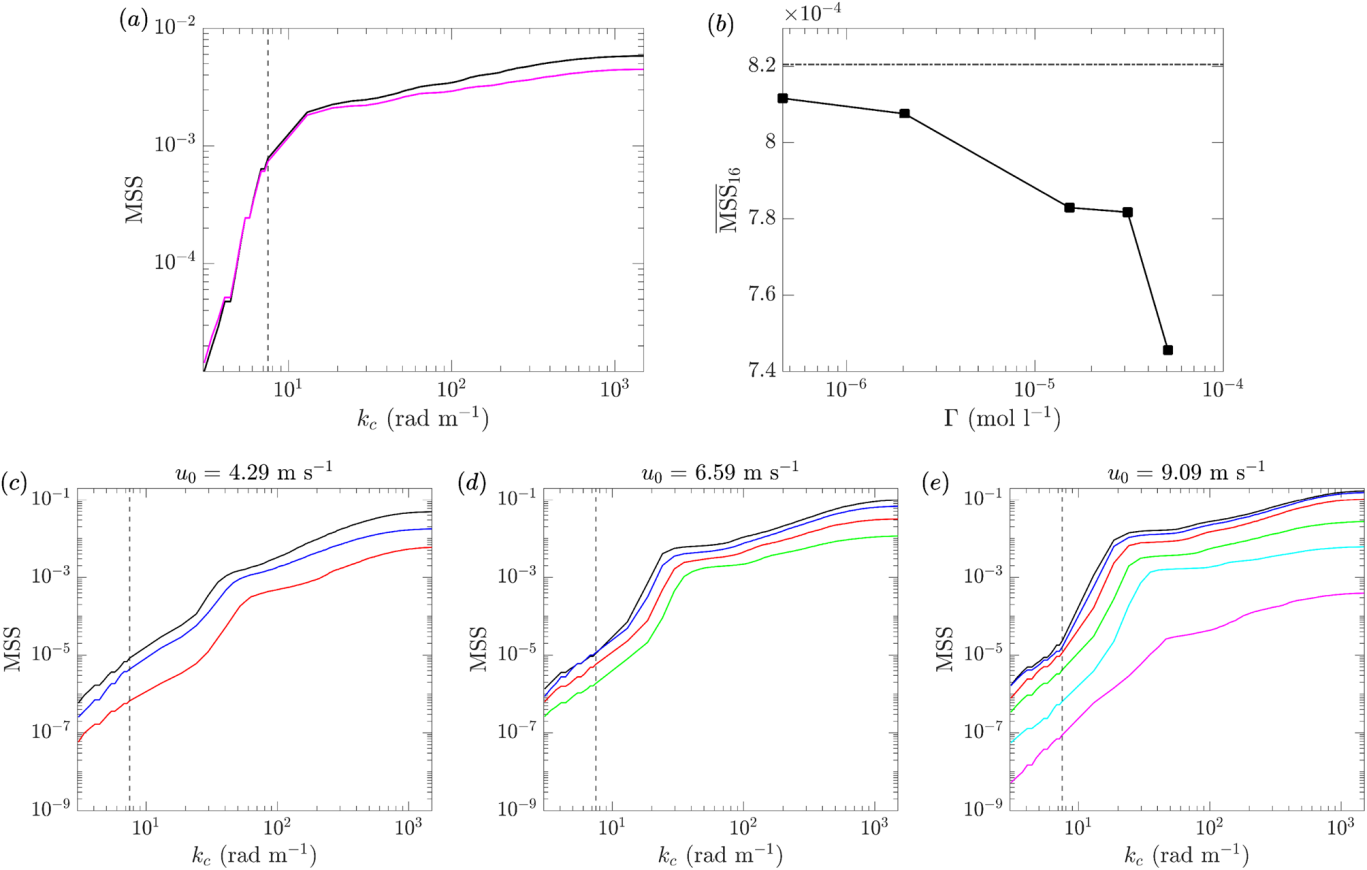
(**a**) MSS as a function of cut-off wavenumber $k_c$ for mechanically generated waves with surfactant concentrations $\Gamma _0$ () and $\Gamma _8$ (); (**b**) $\overline{\text {MSS}}_{16}$ computed with $k_c=7.5$ rad m$^{-1}$ for different concentration levels $\Gamma$ for mechanically generated waves, with result from $\Gamma _0$ marked (- $\cdot$ -); (**c**–**e**) MSS as a function of $k_c$ at reference wind speeds $u_0=4.29$, 6.59, and 9.09 m s$^{-1}$ respectively, with surfactant concentrations $\Gamma _0$ (), $\Gamma _1$ (), $\Gamma _2$ (), $\Gamma _3$ (), $\Gamma _4$ (), and $\Gamma _5$ (). The locations of $k_c=7.5$ rad m$^{-1}$ are indicated by vertical dashed lines in (**a**) and (**c**–**e**).

Furthermore, since the observed wind waves are developed under both effects of wind shear stress and wave damping, with both affected by the presence of surfactants, it is worthwhile to elaborate the physical mechanisms involved in the process. While the Marangoni damping for waves has been discussed in §5.1, the reduced wind shear stress may be physically interpreted as follows: As the wind blows on the free surface, a shear layer is developed in water which tends to transport surfactants downstream. This tendency results in a gradient of surfactant concentration, leading to a reverse Marangoni flow resisting the formation of the shear layer by the wind. This Marangoni process effectively reduces the wind shear stress (which has to be related the flow shear stress at the interface).

Finally, we discuss the implication of the results in surfactant experiments to the MSS anomalies in CYGNSS remote sensing. Figure 22a shows the MSS at sensor 6 as a function of $k_c$ for mechanically generated waves in clean water and with the highest concentration $\Gamma _8$. At CYGNSS cut-off wavenumber $k_c=7.5$ rad m$^{-1}$ indicated in the figure, we see a difference of the two values of MSS which is not significant but larger than the difference in cases of floating particles (Fig. 14). A more quantitative picture is shown in Fig. 22b, which plots the counterpart of Fig. 17a but for $k_c=7.5$ rad m$^{-1}$. Comparing the clean-water result and the result with $\Gamma _8$, we see a reduction of MSS by $O(10\%)$. For wind-wave cases, Fig. 22c–e show the MSS at sensor 6 as a function of $k_c$ for different concentration levels (for which waves are excited). In these figures we see clear difference of MSS even at $k_c=7.5$ rad m$^{-1}$. Quantitatively, with surfactant levels $\Gamma _2$, $\Gamma _3$ and $\Gamma _5$, the MSS is reduced by 8$\%$, 17$\%$ and 50$\%$ relative to the clean-water results for $u_0$=4.29, 6.59 and 9.09 m s$^{-1}$, respectively. Considering the CYGNSS MSS anomaly which is $O(20\%)$ (in terms of reduction from the Katzberg model results), it is clear that the presence of surfactants in wind seas is the most influential factor for this remote sensing application.

## Conclusions and discussions

In this paper, we investigate the physical mechanisms underlying the MSS anomaly measured by CYGNSS which has been used to track oceanic microplastics. For this purpose, we experimentally study the effect of floating particles and surfactants on surface waves (in terms of the energy and surface roughness) generated by a mechanical wave maker and/or wind. The floating particles are tested with two sizes of 0.5 cm and 0.8 cm, and our results show that their damping effect on surface waves critically depends on the area fraction of coverage, irrespective of the sizes. Damping effects on both energy and MSS are only observed for fractions above $O(5\sim 10\%)$, which are much higher than the oceanic microplastics situation of $O(0.1\%)$. For surfactants, experiments with mechanically generated irregular waves show that they generally result in enhanced damping for both the energy and MSS. However, the “optimal” concentration level corresponding to the maximum damping for monochromatic waves cannot be identified for irregular waves tested in our study. In the experiments of waves generated by wind, we find that the presence of surfactants significantly suppresses the wave generation, due to its combined effect of reducing the wind stress and increasing the surfactant-induced damping. The wind stress, obtained from the measured wind profile, is further found to follow an exponential dependence on the surface tension, and a power-law dependence on the concentration level of the surfactant (with the power-law exponent linearly related to the wind speed).

The key results of MSS in this paper are also presented for different cut-off wavenumber $k_c$. When considering the CYGNSS cut-off wavenumber $k_c=7.5$ rad m$^{-1}$, we find that the effect of floating particles on MSS is negligible even at the highest fraction above $10\%$ tested in the current work. The mechanically generated waves with surfactants of highest concentration $\Gamma _8$ result in a $O(10\%)$ reduction relative to the MSS in clean water. In comparison, the wind-waves generated with moderate surfactant concentration of $\Gamma _3$ leads to a $O(17\%)$ reduction from the MSS in clean water, for a wind speed about 9 m s$^{-1}$. Considering the MSS anomalies observed by CYGNSS, which corresponds to CYGNSS MSS $O(20\%)$ lower than the results from the standard Katzberg model, we conclude that the effect of surfactants in a wind sea is a key influential factor for this remote sensing application.

Finally, we remark that the size of the floating particles tested in the current work is limited at the millimeter scales. Further fragmented pieces of plastic in the range of a few microns or smaller, known as nano-particles, may drastically change the flow physics. Due to the particle interactions, e.g., mutually repulsive forces, the nano-particles at the fluid interface (as colloidal plastic) can behave indistinguishably from surfactants. Since nano-plastic is also a source of oceanic plastic pollution^45^, it is of interest to design future experiments to quantify their effect on wave damping for remote sensing applications.

## Data Availability

The data sets supporting the results of this article are included within the article.
